# Colorectal cancer patients’ outcome in correlation with dietary and nutritional status: a systematic review

**DOI:** 10.1080/07853890.2023.2281662

**Published:** 2023-12-19

**Authors:** Rahmawati Minhajat, Tutik Harjianti, Haerani Rasyid, Agussalim Bukhari, Itzar Chaidir Islam, Ahmad Taufik Fadillah Zainal, Andi Muh Aunul Khaliq Gunawan, Arman Caesar Ramadhan, Handayani Hatta, Nurul Izza Syamsu Alam, Sanjaya Winarta, Aldian Irma Amaruddin, Faqi Nurdiansyah Hendra

**Affiliations:** a Hematology and Medical Oncology Division, Internal Medicine Department, Faculty of Medicine, Hasanuddin University, Makassar, Indonesia; b Kidney and Hipertension Division, Internal Medicine Department, Faculty of Medicine, Hasanuddin University, Makassar, Indonesia; c Department of Nutritional Sciences, Faculty of Medicine, Hasanuddin University, Makassar, Indonesia; d Medical Education Department, Faculty of Medicine, Hasanuddin University, Makassar, Indonesia; e Medical Doctor Program, Faculty of Medicine, Hasanuddin University, Makassar, Indonesia; f Department of Parasitology, Faculty of Medicine, Hasanuddin University, Makassar, Indonesia; g Department of Anatomy, Faculty of Medicine, Hasanuddin University, Makassar, Indonesia

**Keywords:** Colorectal cancer, dietary, nutritional status

## Abstract

Colorectal cancer (CRC) is one of the most common cancers worldwide and it involves various biomolecular and cellular levels. CRC has possibly happened due to aging, urbanization, and diet. Different foods have varying effects on the gastrointestinal cells, that’s why additional research is necessary to create effective medical interventions. This review aimed to evaluate the correlation between dietary and nutritional status on the outcome of CRC patients. Study results showed that a healthy diet such as fruit and vegetables is the best diet for improving colorectal cancer outcomes. Moreover, nutritional status affected CRC patients’ outcomes, where high BMI increases the risk of having CRC. However, low BMI was associated with CRC progression and poor quality of life.

## Introduction

Colorectal cancer (CRC) is characterized as a precancerous lesion from the normal colonic and rectal epithelium [1]. CRC is the third most frequent malignancy and the fourth leading cause of cancer mortality. The majority of CRC cases are found in Western nations, and its prevalence is growing annually. The chance of developing colorectal cancer is about 4%–5%, and the risk depends on certain personal characteristics or behaviors such as age, history of chronic diseases and lifestyle [2,3].

The incidence of this disease in the United States of America in 2019 was over 1.5 million people of 776,120 men and 768,650 women. Approximately one-third (35%) of these individuals were diagnosed within five years. More than half (56%) are aged 65–84 years, although the incidence in the young population is increasing annually [4–6]. CRCs are often preceded by a noncancerous proliferation of mucosal epithelial cells. These polyps can develop slowly for ten to twenty years before becoming malignant. The most prevalent histological pattern in advanced stages of cancer is differentiated adenocarcinoma, which accounts for 53.52% of all stages described as grade III or IV [7].

Human development levels differ by tenfold in colorectal cancer incidence and death rates of CRC. This suggests that inequities are expanding, and the burden on nations in transition increases [8]. In 2020, there were 17,368 cases of CRC in Indonesia, with 9444 deaths [9]. Meanwhile, the survival rate for CRC is concerning 64% cases in five years but only 12% for poor grade CRC cases. More study is still needed to develop sensible approaches for medical preventive and rehabilitative development [10]. A country’s quality of care and the availability of a population screening program both have an impact on the stage distribution at diagnosis, which in turn affects mortality rates [11].

CRC may develop through various mechanisms, including chromosomal instability (CIN), microsatellite instability (MSI), and inflammatory pathways [12]. A study by Yusuf et al. found that CRC was associated with a mutation on chromosome 15 in the Indonesian population [13]. The CRCs that develop into the colon or rectum wall can penetrate blood or lymphatic vessels, permitting metastasis to remote organs thru the blood or close by lymph nodes [14].

CRC was possibly due to aging, urbanization, increased alcohol consumption, obesity, smoking, and suboptimal diet [15]. Diet may have a role in the development of CRC either negatively or positively, since it substantially influences the colon’s microbiome, with various foods having varying impacts on the microflora population and intestinal inflammation [14]. Inappropriate dietary patterns reported could be increase CRC risk [16].

Supplements that give a greater level of individual nutrient intake than the diet provide an alternate option for the nutritional prevention of colorectal cancer (chemoprevention) [17]. Protective factors associated with a decrease in the incidence of CRC include regular physical activity, a diet rich in fruits and vegetables, garlic, high fiber diet, folate-rich diet, Calcium, Dairy products, Vitamin D, and Vitamin B6, magnesium intake, and fish consumption. However, increasing red or processed meat consumption may raise the chance to become CRC [18–21]. Numerous studies are required to conclusively establish the advantages and hazards of folate on colorectal cancer incidence and death rates [22]. The data on body size (weight, height, and BMI) indicated that higher weight (women only) and height (both genders) were related to an increased risk of colorectal cancer, but not BMI [23].

This review purposed to evaluate the relevance between dietary and nutritional status to colorectal cancer patients and provide recommendations for doctors and patients to carry out preventive and curative diet and nutrition programs in CRC patients.

## Method

### Literature search

A literature search undertaken from multiple databases, including PUBMED, MEDLINE, and EMBASE, for this systematic review. This will be conducted using the keywords of (((colorectal cancer) OR (bowel cancer) OR (colon cancer) OR (rectal cancer) OR (colorectal carcinoma) OR (bowel carcinoma) OR (rectal carcinoma)) AND ((nutrition) OR (body mass index) OR (nourishing) OR (nourishment))). Furthermore, numerous valid research from outside the database will be added when the conditions are met.

### Study eligibility and screening criteria

The study criteria for inclusion in this systematic review are (1) The design is an observational study; (2) The language used is Indonesian or English; (3) Nutritional status, dietary intake, or supplementation as a source of exposure; (4) Evaluated Incidence, recurrence, complication mortality, and quality of life in CRC patients. The following research requirements were be disregarded: (1) No abstract; (2) Unavailable access for Full-text; and (3) Incorrect article type. Studies of the literature that meet the eligibility requirements were included, while the others were eliminated with further explanation. The conflicts arising from combining the research were considered together until a conclusion is reached. The study was conducted according to PRISMA guidelines.

### Data collection

Data grouping were conducted for (1) Main author; (2) Year of publication; (3) Research location; (4) Design of the study; (5) Sample sizes; (6) Mean/Range age; (7) Exposure Type; (8) Outcome. Data collection was conducted by RM, AB, AC, SW, H, NI and then cross-checked by TH, HR, AT, AIA, FNH, AMA, and IC. The author is contacted when included research has missing data; however, the research is eliminated when answers are not given.

### Quality assessment

Five reviewers consisting of AT, AM, IC, SW, and AC assessed the quality of the observational research using the Newcastle-Ottawa Scale (NOS) [24].

In a cross-sectional and case-control study, an evaluation became completed on 3 major aspects: selection, comparability, and exposure. Meanwhile, in a cohort study, the exposure issue became changed with an evaluation of the study result. The evaluation is conducted through a point-by-point discussion. The Cochrane risk of bias tools examined the randomized controlled trial [25] across seven domains. (1) Random Sequence Generation; (2) Allocation concealment; (3) Blinding of contributors and personnel; (4) Blinding of final results assessment; (5) Incomplete final results statistics (6) Selective reporting (7) Additional biases. Each domain will be assigned a low, high, and unclear risk score. In addition, the results of quality assessments from RCT studies are reported in graphical form.

## Results

### Literature search and screening

**Figure 1.**
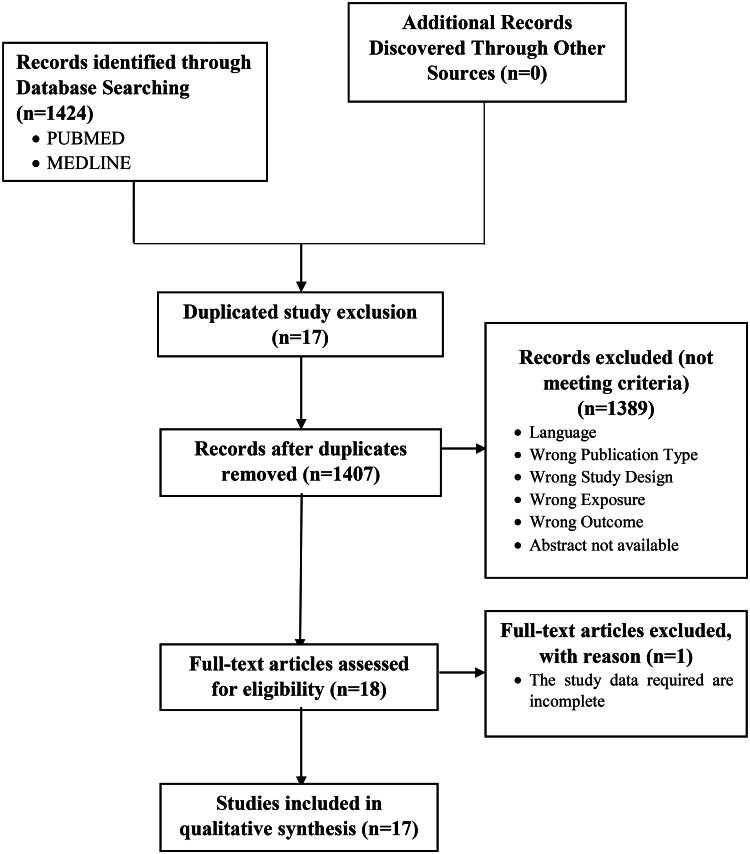
Preferred reporting items for systematic reviews.

In this systematic review, the keywords obtained from 1424 studies were filtered using predetermined criteria after searching PUBMED and MEDLINE. Prior to the screening, 17 duplicated studies were removed. In addition, 1407 research papers’ titles and abstracts were evaluated by three independent reviewers (RM, SW, IC). A total of 1389 studies were removed because they did not meet predefined criteria. The next 18 were screened by thoroughly reading the text. As a result, one study was removed due to an incomplete result, leaving 17 studies with a total sample size of 30.482 that met the criteria and were considered for qualitative analysis. Figure 1 depicts the entire set of search and filter results.

### Characteristics of the eligible studies

The 17-research considered were primary investigations done in 13 different countries and included a total sample size of 30,482 participants. Ten of the seventeen studies used a cohort design, and three employed a case-control design, one employed a cross-sectional design, and three employed an intervention design. Furthermore, seven research looked at the influence of nutritional status on colorectal cancer, while 12 looked at the effect of food on colorectal cancer. Table 1 summarizes the features of the included studies in detail.

**Table 1.** Characteristics of inclusion studies.

No	First Author (Year)	Year	Country	Study Design	Sample size	Mean Age/range	Exposure	Outcome
1	Shiao	2018	United states	Case control study	106 participants	54	Healthy eating practices	Progression of colorectal cancer
2	Kurk	2019	Netherlands	Cohort study	625 participants	65.8	Body mass index (BMI) and Skeletal muscle index (SMI)	Colorectal cancer progression
3	Barrubes	2018	Spain	Cohort study	7447 participants	Male: 55–80; Female: 60–80	Dairy product	Risk of colorectal cancer
4	Formica	2021	Rome	Cohort study	173 participants	44–88	Body mass index (BMI)	Quality of life patients with colorectal cancer
5	Lee	2020	Seoul	Cohort study	3449 participants	65 years	Body mass index (BMI)	Colorectal cancer reccurence and survival
6	Lin	2017	China	Randomized Controlled Trial	110 participants	52.61	Healthy diet	Progression of colorectal cancer
7	Guinter	2020	Canada	Cohort study	2498 participants	69.9	Body mass index (BMI)	Colorectal cancer incidence
							Healthy diet	Progression of colorectal cancer
8	Gathirua-Mwangi	2017	Spain	Cross sectional study	4500 participants	57.3	Body mass index (BMI) and waist circumference (WC)	Risk of having an advanced colorectal neoplasia (AN)
9	Mehta (2017)	2017	United States	Cohort study	3260 participants	Male: 40–75; Female:30–55	Western and prudent dietary patterns	Risk of Colorectal cancer
10	Gigic	2018	United States	Cohort study	192 participants	62	Western diet, fruit & vegetable diet, bread & butter diet, and high-carb diet	Quality of life of patients with colorectal cancer
11	Feliciano	2017	United States	Cohort study	2290 participants	50–79	Body mass index (BMI)	Colorectal cancer incidence
12	Fruge	2021	United States	Randomized Controlled Trial	50 participants	48	Green leafy vegetables (GLV) diet	Risk of colorectal cancer
13	Obon-Santacana	2019	Spain	Case control study	1852 participants	66.8	Pro-inflammatory diet	Risk of colorectal cancer
14	Wesselink	2021	Netherlands	Cohort study	1478 participants	66.2	Habitual dietary intake	Colorectal cancer reccurrence
15	Lopez-Rodriguez-Arias	2021	Spain	Randomized Controlled Trial	156 participants	69.5	Postoperative suplementation periOlimel N4-E	Postoperative complication and length of hospital stay Postoperative colorectal cancer complications and length of hospital stay
							Body mass index (BMI) and Skeletal muscle index (SMI)	
16	Alegria-Lertxundi	2020	Spain	Case control study	308 participants	61.5	Food group consumption and diet quality	Colorectal cancer incidence
17	Baar	2020	Netherlands	Cohort study	1988 participants	Male: 68; Female: 67.8	Skeletal muscle index (SMI), subcutaneus and visceral adipose tissue	Colorectal cancer mortality

### Quality assessment result

The quality of the 14 included observational studies was represented by the total number of stars obtained for each study ranging from 0 to 9. For example, the lowest score in one cross-sectional study was four stars; the average score in six studies was six stars; and the highest score in two studies was eight stars. Table 2 contains the exact data from the quality assessment.

**Table 2.** Quality assessment result using Newcastle-Ottawa Scale.

Study	S				C	E/O			SUM
	1	2	3	4	1	1	2	3	
Shiao (2018)	*	*	0	*	**	0	*	*	7
Kurk (2019)	*	*	*	*	**	*	*	–	8
Barrubes (2018)	*	*	*	*	–	*	*	–	6
Formica (2021)	–	–	–	*	**	*	*	*	6
Lee (2020)	*	*	*	*	–	*	*	–	6
Guinter (2020)	*	*	–	*	**	*	*	*	8
Gathirua-Mwangi (2017)	*	*	0	0	0	*	*	0	4
Mehta (2017)	*	*	–	*	*	*	*	–	6
Gigic (2018)	*	*	–	*	*	*	*	–	6
Feliciano (2017)	*	*	*	*	*	*	*	–	7
Obon-Santacana (2018)	*	*	0	*	**	0	*	0	6
Wesselink (2021)	*	*	–	*	*	*	*	–	6
Alegria-Lertxundi (2020)	*	*	*	*	**	0	*	0	7
Baar (2020)	*	*	*	*	*	*	*	–	7

**Figure 2.**
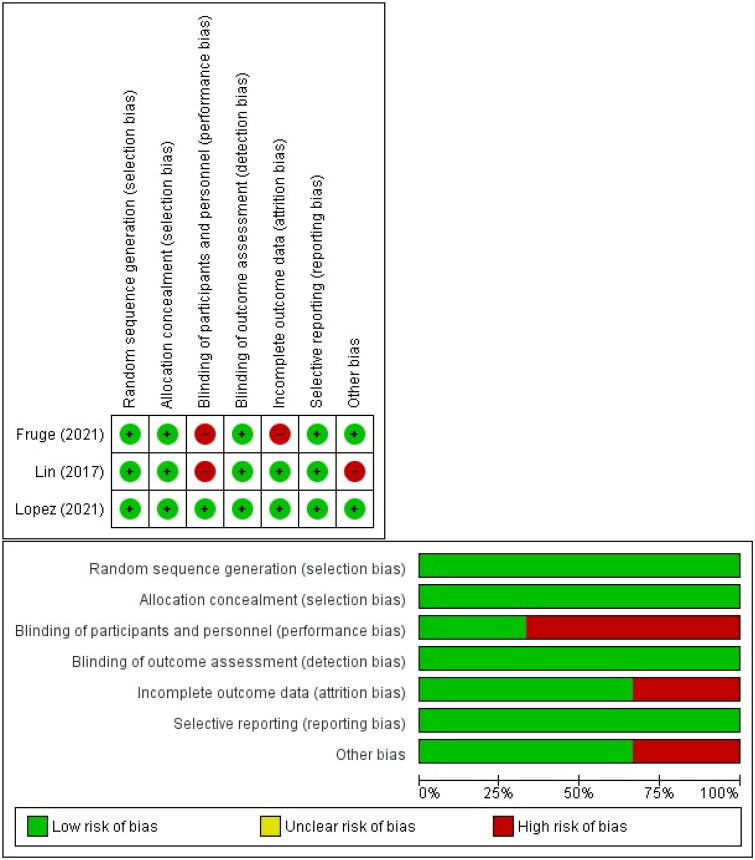
Quality assessment result using Cochrane risk of bias tools.

The quality of three randomized controlled trials was assessed using graphs and a risk of bias summary. In terms of participant blinding, two studies had a significant risk of bias. This is due to the fact that patients are aware of the intervention delivered, and the outcome measured is subjective; hence, there is a significant possibility of bias. One additional research, however, found a low risk of bias over the whole domain, and Figure 2 displays the complete breadth of the quality evaluation.

## Discussion

Nutritional status has a significant impact on the occurrence and progression of colorectal cancer. Ten studies assess the role of nutritional status in colorectal cancer for various outcomes [26–33]. Five studies evaluate the nutritional status from the body mass index (BMI) aspect [26–31]. Studies conducted by Guinter et al. and Feliciano et al. assessed the impact of BMI on colorectal cancer incidence [26,27]. Those studies showed that increased BMI was strongly associated with colorectal cancer incidence. The increase in BMI indicates excess body fat, which can impair the body metabolic system and be colorectal cancer. The Feliciano et al. study also assessed the impact of BMI on colorectal cancer mortality. This study reported that BMI ≥ 35 kg/m^2^ was associated with higher cancer mortality rates with HR 1.40 and 95%CI 1.04–1.88. Meanwhile, a study conducted by Kurk et al. reported that the loss in BMI was associated with early colorectal cancer progression but was not associated with late disease progression [28]. The study examines the incidence, course of illness and the effect of BMI on the quality of life of colorectal cancer patients. Furthermore, Formica et al. reported that BMI was strongly associated with the quality of life of colorectal cancer patients, where BMI < 23 kg/m^2^ was associated with a poor score of health-related quality of life (HRQoL) [29].

The study conducted by Lee et al. assessed the Impact of BMI on colorectal cancer patient recurrence and survival. This study showed that BMI or even body weight was not associated with colorectal cancer recurrence and survival. A large cohort study is recommended to validate these findings because the results of Lee et al. conflict with previous conclusions that BMI or body weight was connected with colorectal cancer recurrence and survival [30]. This systematic review also includes research that looked at the effect of BMI on the chance of developing advanced colorectal neoplasia (AN), which is a combination of colorectal cancer and advanced precancerous polyps. The study conducted by Gathirua-Mwangi et al. showed that obese BMI (≥30 kg/m^2^) was associated with AN (OR 1.87 and 95%CI 1.08–3.23). However, this study found that waist circumference (WC) rather than BMI was superior for predicting AN risk, where participants with increased WC (OR 1.44 and 95%CI 1.08–3.23) or maintained a high risk of WC (≥35 inches for females and ≥40 inches for males; OR 2.5 and 95% CI 1.08–3.23) had an increased risk of AN. According to the findings of the Gathirua-Mwangi et al. study, having an unhealthy BMI and WC may increase the likelihood of having AN [31].

Three studies in this systematic review also assessed the nutritional status from different aspects together with BMI [28,32,33]. The study conducted by Kurk et al. also assessed the nutritional status from the skeletal muscle index (SMI) aspect and its impact on the disease progression. SMI loss can occur without weight loss and may be key in identifying early protein-energy malnutrition in colorectal cancer. This study reported that the loss in SMI was associated with late colorectal cancer progression. Another study conducted by López-Rodríguez-Arias et al. reported that poor body composition of low SMI and high BMI ≥ 35 kg/m^2^ was strongly associated with significant postoperative colorectal cancer complications and the increase in length of hospital stay for 3.6 d [32]. The last study conducted by Baar et al. assessed the nutritional status from SMI, subcutaneous and visceral adipose tissue (SAT and VAT, respectively) aspects and its impact on colorectal cancer mortality. SMI and mortality were strongly associated in men, whereas lower SMI level was associated with higher mortality. Lower SAT level, on the other hand, was substantially connected with increased mortality in both men and women, although VAT level was not significantly associated with mortality. There are still many limitations, one of which is that there are several essential outcomes affected by nutritional status in colorectal cancer like the stadium and functional capacity yet to be investigated. Therefore, further future research to assess the various outcomes affected by the nutritional status of colorectal cancer is still needed [33].

Furthermore, dietary exposure also plays an essential role in the development of colorectal cancer. Several studies have indicated that various diets affect colorectal cancer. Patients with a healthy diet can prevent the progression of colorectal cancer [34]. This is supported by the study of Guinter et al. and Lin et al. where the deterioration of colorectal cancer can be prevented with a healthy diet by controlling glucose hemostasis to maintain the patient’s energy balance as well as increase body weight, albumin and prealbumin [35].

Healthy diets that improve colorectal cancer outcomes are rich in fruits, vegetables, fish, poultry, and whole-grain products [36,37]. These results are consistent with the findings of Gigic et al. who focused on the use of fruits and vegetable diets to improve the quality of life of patients with colorectal cancer [38]. In another finding by Fruge et al. a randomized controlled trial was used to determine the effects of green leafy vegetables including spinach, kale, collards, mustard greens, and turnip greens in colorectal cancer patients. The result showed the ability to repair DNA damage as evidenced by decreased TNFα and increased vitamin K from plasma and fecal 8OHdG samples [39]. Therefore, the fruit and vegetable diet is the most favorable candidate for a healthy diet in this finding. A diet rich in fruits and vegetables has been shown to prevent and slow the growth of colorectal cancer because it is rich in fiber, which is considered a protective agent in preventing constipation [38]. Green leafy vegetables also inhibit cytotoxic and carcinogenic activities in heme [39]. Additional diet using supplements such as periOlimel N4-E also reported improved patient outcomes, particularly in postoperative and length of hospital stay [32]. Therefore, a healthy diet such as fruits & vegetables and supplementation is a type of diet that is beneficial for colorectal cancer patients. In the future, it is also possible that the combination of the two can provide a better outcome, so more research is needed.

Some pro-inflammatory diets include more red and processed meat, extra sugar and refined carbohydrates, a bread and butter diet, and milk/dairy products. Dietary fat cheeses were reported to increase the risk of colorectal cancer in the studies of Mehta et al.; Gigic et al.; Wesselink et al. and Alegria et al. [36–38,40,41]. The Western diet is poor in fiber and contributes to the carcinogenic knockdown pathway, which is defined by particular molecular modifications including CIMP-high, MSI-high, and BRAF mutations [36]. The bread and butter diet is related to continuing appetite loss, and new findings indicate that appetite reduction at the start of follow-up is strongly associated with lower survival [38]. Milk and dairy products are heavy in saturated fat, and some studies indicate that consuming a high-fat diet increases bile acid excretion. An increase in bile acid concentrations above physiological levels has promoted colorectal cancer [37]. However, Wesselink et al. did not show a relationship between an anti-inflammatory diet and the progression of colorectal cancer, as was predicted according to the short duration of patient follow-up of 2.8 years compared to another study from 5 years to 12 years [42].

## Conclusion

A healthy diet such as fruit and vegetable improve colorectal cancer outcomes. Meanwhile, other dietary patterns, such as the western diet, bread and butter, and milk/dairy products, have increased colon cancer risk. Nutritional status affects some colorectal cancer outcomes, where a high BMI increases the risk of having colorectal cancer, and a low BMI is associated with disease progression and poor quality of life. Poor body composition raises the likelihood of significant postoperative colorectal cancer complications and duration of stay. SMI and SAT levels are both linked to colorectal cancer mortality.

## Data Availability

The authors confirm that the data supporting the findings of this study are available within the article.
